# Commentary: The Effects of High Intensity Interval Training vs Steady State Training on Aerobic and Anaerobic Capacity

**DOI:** 10.3389/fphys.2016.00495

**Published:** 2016-10-25

**Authors:** Victor S. Coswig, Paulo Gentil, João P. A. Naves, Ricardo B. Viana, Charles Bartel, Fabrício B. Del Vecchio

**Affiliations:** ^1^Department of Physical Education, Superior School of Physical Education, Federal University of PelotasPelotas, Brazil; ^2^Department of Physical Education, School of Physical Education and Dance, Federal University of GoiásGoiânia, Brazil

**Keywords:** physiology exercise, circuit-based exercise, interval exercise, high-intensity, exercise tests

The research article by Foster et al. (2015) aimed to compare high intensity interval training (HIIT) protocols with steady state exercise and conclude that HIIT protocols are not superior to conventional exercise training in sedentary young adults. We would like to compliment the authors for the interesting work and findings, however, it is necessary to point out some relevant issues, especially regarding protocols configuration and interpretation of the results.

Despite the worldwide popularity of Tabata's protocol, it is necessary to be more critical about its use and adaptation. Foster et al. (2015) cited Tabata et al. (1996) for the very brief, very high intensity interval training used in the study. However, they actually described a protocol similar to the one published by Tabata et al. (1997), which should be performed until exhaustion with only 5–6 bouts at 170% of the VO2max, and not 8 as used in the study. The original protocol, published in 1996, proposed 7–8 sprints at a constant load performed until the pedaling frequency dropped below 85 rpm. Load was incremented when the participants could perform more than 9 sets, and not only based on RPE decrements.

The Meyer et al. (1990) interval training protocol used by Foster et al. (2015) also did not follow the original description. The original prescription was based on maximum heart rate (86 ± 3% of maximum), with 1-min intervals and an effort:pause ratio of 1:1, while the protocol described by Foster et al. (2015) was based on power output (PO), with 30-s intervals and an effort:pause ratio of 1:2.

In our opinion, researchers should exercise caution when attempting to replicate previously used protocols, since deliberate changes to these original parameters used will most likely yield different results. In addition, the divergence between the used protocols from the protocols cited in the references may cause difficulties when replicating the study. Therefore, authors should be more meticulous in indicating these changes and their consequent limitations. Moreover, one of the major problems with HIIT studies is the wide range of training protocols utilized across studies, limiting more conclusive inferences. In this way the modification of existing protocols in subsequent studies does not contribute positively to increase our comprehension about HIIT prescription.

Furthermore, based on our practical experience and theoretical evidence, it seems unrealistic to perform 8 sprints at a speed of 90 rpm at 170% of the aerobic power, especially in sedentary individuals. Experiments in our laboratories indicate that a reasonable intensity for reproducing the original Tabata Protocol should use ~120% of the maximal aerobic power. Power output reductions were already evident after 8-s sprints with 15-s rest (Billaut et al., 2003). After ~10-s sprints it takes 120-s to recover the initial power production (Cooke and Barnes, 1997). How could we expect the maintenance of 170% of peak aerobic power after 20-s sprints for 8 repetitions, with only 10-s pause in previously sedentary subjects?

Another limitation is related to enjoyment issues. To our understanding, enjoyment values do not make fair assessments when: (i) original protocols are not really followed; and (ii) only acute measurements are performed. It appears to be obvious that after 4-min of very intensive exercise an enjoyment scale should indicate a low value. Therefore, in order to adequately address the question, researchers should consider long-term adherence and/or intention to engage in the exercise program (McRae et al., 2012; Jung et al., 2014). Moreover, even though the Tabata protocol is reported as less enjoyable, one cannot forget that it was more time efficient than the other two training models. The occurrence of similar gains in all measured variables with a training volume five times smaller is extremely relevant, since lack of time is the main reported reason for being physically inactive (Trost et al., 2002; Gibala et al., 2012).

It is time to give careful consideration to research studies focused on HIIT. In our opinion, there is a serious problem in replicating HIIT protocols, and this is a direct result of deliberate protocol adaptations, along with improper descriptions of the methods used during the experiments, as well as inadequately referencing bibliographic citations. Our concern is that this could lead to significant mistakes in both the scientific and practical applications of HIIT, which can potentially result in undesirable outcomes. Especially as the results obtained by these adapted protocols can vary greatly from the results obtained in the original studies. Finally, time efficiency, motivation, and health/fitness improvements associated with HIIT potentially exceeds the acute negative effects of its high intensity, and this should be considered when discussing its cost effectiveness (Del Vecchio et al., 2015).

## Author contributions

VC, JN, RV, PG, CB, and FD: Conception, drafting the article, revising it critically, and final approval of the version to be published.

### Conflict of interest statement

The authors declare that the research was conducted in the absence of any commercial or financial relationships that could be construed as a potential conflict of interest.
